# Pharmacometabolomics reveals urinary diacetylspermine as a biomarker of doxorubicin effectiveness in triple negative breast cancer

**DOI:** 10.1038/s41698-022-00313-4

**Published:** 2022-10-07

**Authors:** Thomas J. Velenosi, Kristopher W. Krausz, Keisuke Hamada, Tiffany H. Dorsey, Stefan Ambs, Shogo Takahashi, Frank J. Gonzalez

**Affiliations:** 1 Laboratory of Metabolism, Center for Cancer Research, National Cancer Institute, National Institutes of Health (NIH), Bethesda, MD USA; 2 Faculty of Pharmaceutical Sciences, University of British Columbia, Vancouver, BC Canada; 3 Laboratory of Human Carcinogenesis, Center for Cancer Research, National Cancer Institute, National Institutes of Health (NIH), Bethesda, MD USA

**Keywords:** Breast cancer, Cell growth

## Abstract

Triple-negative breast cancer (TNBC) patients receive chemotherapy treatment, including doxorubicin, due to the lack of targeted therapies. Drug resistance is a major cause of treatment failure in TNBC and therefore, there is a need to identify biomarkers that determine effective drug response. A pharmacometabolomics study was performed using doxorubicin sensitive and resistant TNBC patient-derived xenograft (PDX) models to detect urinary metabolic biomarkers of treatment effectiveness. Evaluation of metabolite production was assessed by directly studying tumor levels in TNBC-PDX mice and human subjects. Metabolic flux leading to biomarker production was determined using stable isotope-labeled tracers in TNBC-PDX ex vivo tissue slices. Findings were validated in 12-h urine samples from control (*n* = 200), ER+/PR+ (*n* = 200), ER+/PR+/HER2+ (*n* = 36), HER2+ (*n* = 81) and TNBC (*n* = 200) subjects. Diacetylspermine was identified as a urine metabolite that robustly changed in response to effective doxorubicin treatment, which persisted after the final dose. Urine diacetylspermine was produced by the tumor and correlated with tumor volume. Ex vivo tumor slices revealed that doxorubicin directly increases diacetylspermine production by increasing tumor spermidine/spermine N^1^-acetyltransferase 1 expression and activity, which was corroborated by elevated polyamine flux. In breast cancer patients, tumor diacetylspermine was elevated compared to matched non-cancerous tissue and increased in HER2+ and TNBC compared to ER+ subtypes. Urine diacetylspermine was associated with breast cancer tumor volume and poor tumor grade. This study describes a pharmacometabolomics strategy for identifying cancer metabolic biomarkers that indicate drug response. Our findings characterize urine diacetylspermine as a non-invasive biomarker of doxorubicin effectiveness in TNBC.

## Introduction

Women have a 1-in-8 chance of developing breast cancer, which is the second leading cause of cancer-related death^1^. Triple-negative breast cancer accounts for 10-20% of breast cancers^2^. In the absence of targets to exploit with therapy, TNBC patients are treated with aggressive chemotherapy as the primary systemic treatment. TNBC chemotherapy regimens typically contain a combination of taxanes, cyclophosphamide, and anthracyclines such as doxorubicin, to maximize therapeutic response. Only 30–40% of patients with TNBC who receive taxane- and anthracycline-based therapy will achieve a pathological complete response^3^. TNBC has the worst prognosis of all breast cancer subtypes, with the highest 5-year mortality across all disease stages^4^. Recurrence is frequent in TNBC patients with most events occurring within 3 years of the disease diagnosis^5^. Current techniques for detecting TNBC recurrence rely on imaging and are limited by many factors including tumor size, individual breast characteristics, skill of the examiner, and follow-up to minimize false negatives^6^. Consequently, a recurrence can only be detected months after primary therapy. Therefore, identification of clinical biomarkers that can be used to evaluate drug response during treatment would be beneficial to patients with TNBC.

Metabolites can provide a reliable prognostic and diagnostic readout for disease status^7^. By extending the use of metabolic biomarkers to pharmacometabolomics, metabolites excreted from tumors can be measured by routine non-invasive sampling before and throughout the duration of drug treatment to monitor therapeutic effectiveness. Indeed, properly powered clinical metabolomics studies require many subjects and lack the ability to identify the source of biomarker production. The development of patient-derived xenografts (PDX), which can be grown and passaged for multiple generations while retaining their genetic and molecular integrity, provides a well-suited model to evaluate pharmacometabolomic biomarkers.

In this study, we employed TNBC-PDX models known to be sensitive or resistant to doxorubicin as an innovative pharmacometabolomics approach to identify and characterize urinary metabolic biomarkers of doxorubicin effectiveness. With this approach, we identified diacetylspermine, a catabolic product in the polyamine pathway, as a urinary metabolic biomarker that increases with increasing tumor size, and was produced by the implanted tumor. Using tumor ex vivo tissue slices and applying stable isotope tracer substrates for the polyamine pathway, we further show that the effect of doxorubicin on SAT1 expression and its activity is the mechanism of increased diacetylspermine. Finally, these observations were extended to human breast cancer by showing that urinary diacetylspermine levels are associated with tumor size in a large clinical study and that tumor diacetylspermine is elevated in tumor tissue compared to adjacent noncancerous tissue.

## Results

### Diacetylspermine is a urine metabolic biomarker of doxorubicin effectiveness

**Fig. 1:**
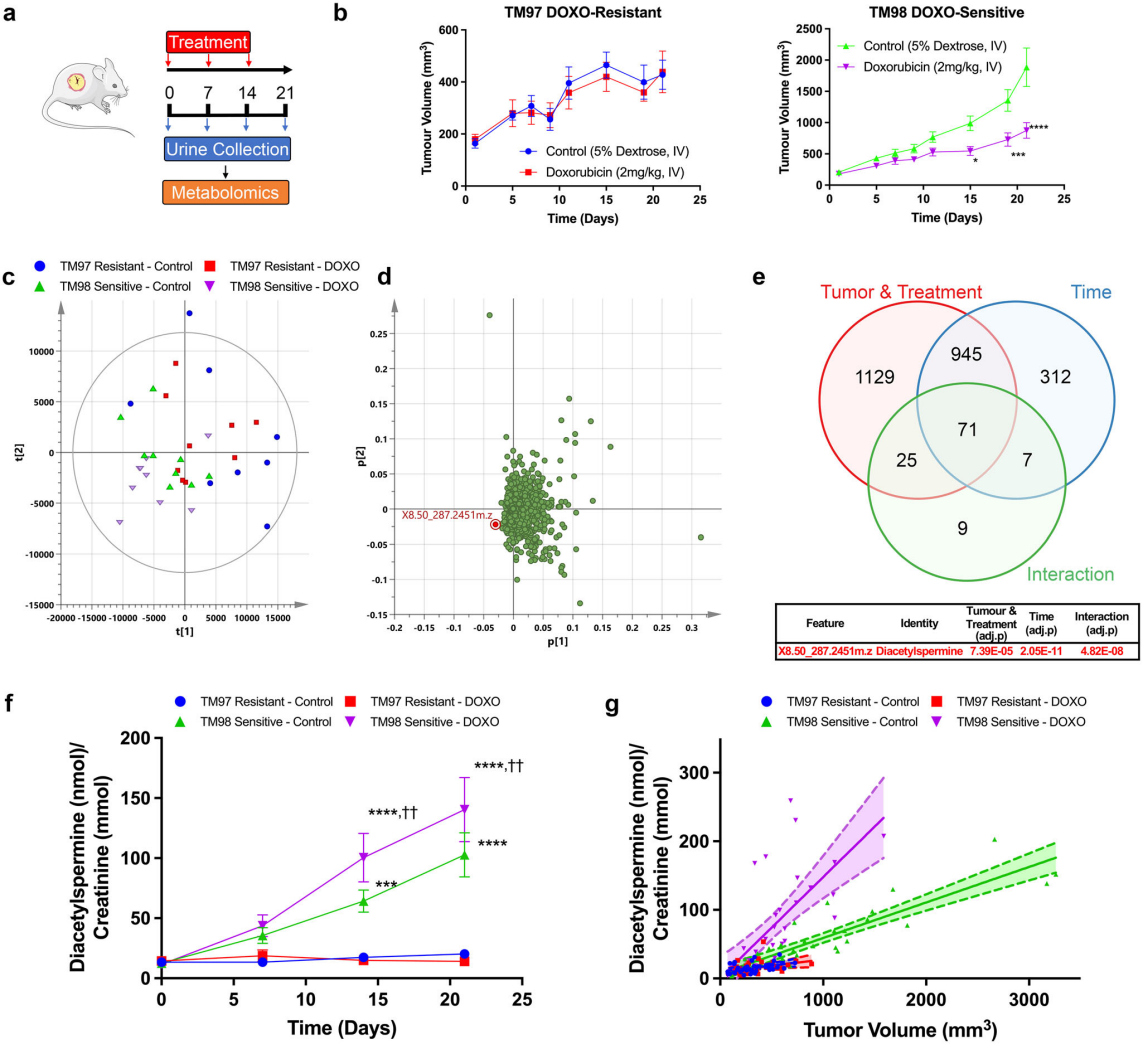
Longitudinal urine metabolomics identifies diacetylspermine as a metabolic biomarker of doxorubicin effectiveness. **a** Experimental design for urine collection of TNBC-PDX TM97 and TM98 mice treated with vehicle (5% dextrose, IV) or doxorubicin (DOXO, 2 mg/kg, IV). **b** Tumor growth curves showing TM97 resistance and TM98 sensitivity to DOXO treatment. **c**, **d** Principal component analysis and loadings plot of untargeted urine metabolomics on day 21. **e** Venn diagram showing number of significantly altered features with X8.56_287.2451 m.z. being the most significant by two-way repeated measures ANOVA. Tumor refers to the tumor identity (TM97 and TM98) while treatment refers to vehicle or doxorubicin. **f** Quantified urinary diacetylspermine and **g** correlation with tumor volume. Data are presented as mean ± s.e.m. or individual values, ***P < 0.001, ****P < 0.0001 vs TM97, ^††^*P* < 0.01 vs TM98 control; *n* = 8–9. Significance was determined by one-way ANOVA with Tukey’s test or two-way repeated measures ANOVA with Sidak’s correction. Correlation plots display 95% confidence intervals. Graphics were generated using Servier Medical Art (smart.servier.com).

To identify metabolic biomarkers of doxorubicin effectiveness, TM97 (doxorubicin-resistant) and TM98 (doxorubicin-sensitive) TNBC-PDX mice were randomly divided and treated with intravenous vehicle or doxorubicin (2 mg/kg) once weekly followed by 24-hour urine collection in metabolic cages. A final urine collection was obtained in the absence of drug treatment to limit drug metabolite interference during metabolomics analysis (Fig. 1a). Doxorubicin treatment attenuated tumor growth in the TM98 TNBC-PDX model, but had no effect on TM97, confirming sensitivity and resistance, respectively (Fig. 1b). Principal component analysis using the metabolome data from day 21 urine samples showed separation of tumor-bearing animals by doxorubicin treatment in TM98, which was partially explained by accumulation of one metabolite, diacetylspermine (Fig. 1c, d). Univariate statistical analysis revealed diacetylspermine as the most significantly altered urine metabolite as a product of the interaction of tumor (TM97 and TM98) & treatment (vehicle and doxorubicin) and the time course (Fig. 1e, Supplementary Table 1). Urine diacetylspermine levels were significantly increased in mice implanted with TM98 when compared to TM97. Interestingly, doxorubicin significantly increased TM98 urinary diacetylspermine levels, despite the corresponding decrease in tumor size during treatment. From day 1 to 21, urine diacetylspermine increased 8.5-fold and 12.8-fold in TM98 mice following treatment with vehicle or doxorubicin, respectively (Fig. 1f). In TM97, urine diacetylspermine was not significantly altered throughout tumor growth and regardless of treatment. Tumor volume was correlated with urine diacetylspermine with a particularly strong correlation for TM98 control-treated mice (*r*^2^ = 0.8, *P* < 0.001, Fig. 1g).

### Doxorubicin-mediated induction of SAT1 increases tumor diacetylspermine production

**Fig. 2:**
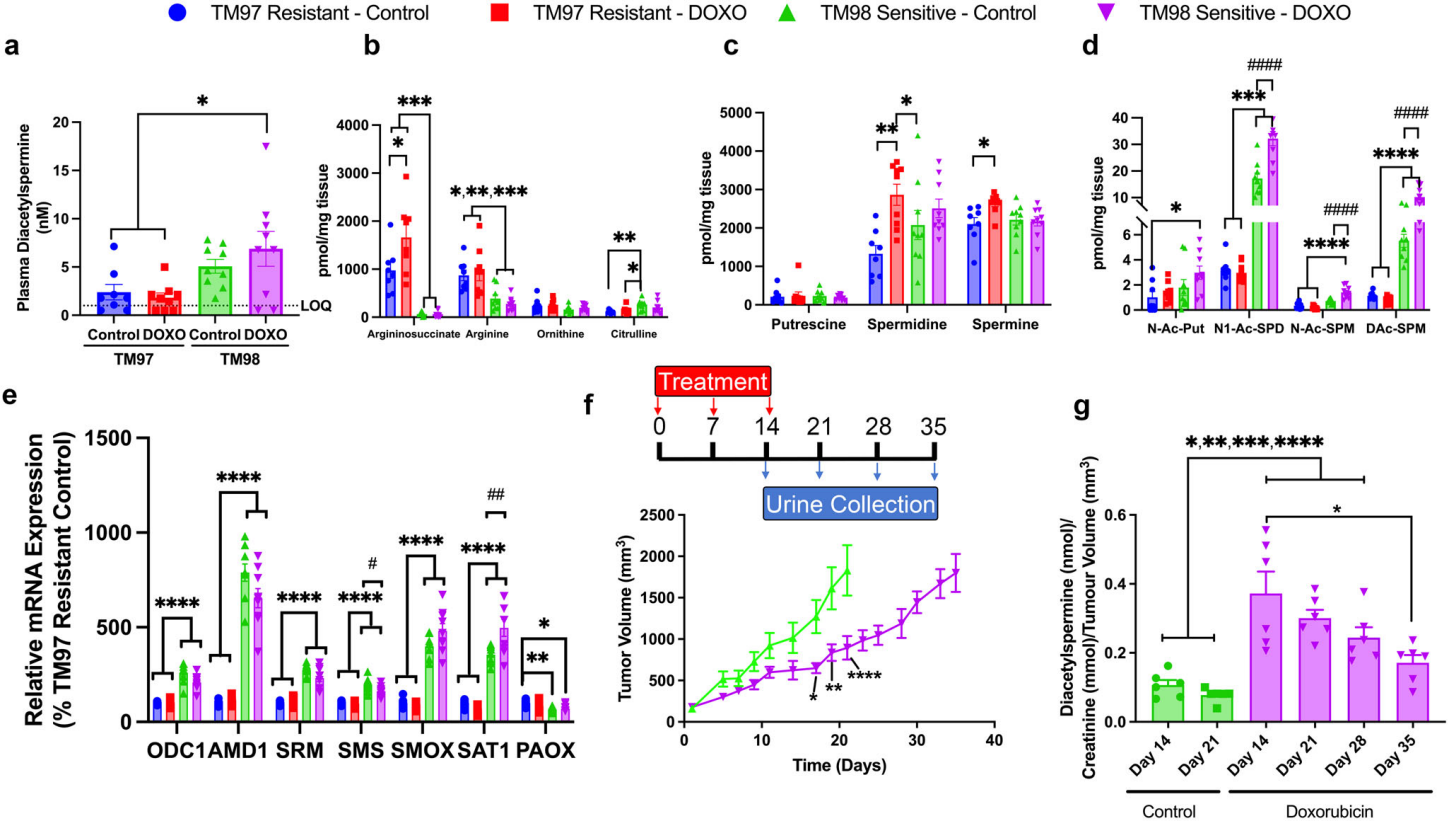
Doxorubicin treatment increases SAT1 expression and tumor production of diacetylspermine, which is sustained following the final dose. **a** Plasma diacetylspermine and tissue urea cycle (**b**), polyamine (**c**) and acetylated polyamine (**d**) metabolites from TM97 and TM98 TNBC-PDX mice treated with vehicle (5% dextrose, IV) or doxorubicin (DOXO, 2 mg/kg, IV). **e** mRNA expression of polyamine pathway enzymes in TM97 and TM98 TNBC-PDX mice treated with vehicle or doxorubicin. **f** TM98 mice treated with vehicle or doxorubicin and placed in metabolic cages for urine collection as indicated. **g** Urine diacetylspermine normalized to tumor volume. Data are presented as mean ± s.e.m. *n* = 8–9 (**a**–**e**), *n* = 6 (**f**, **g**). Significance was determined by one-way ANOVA with Tukey’s test or two-way repeated measures ANOVA with Sidak’s correction, *P < 0.05, **P < 0.01, ***P < 0.01, ****P < 0.0001 vs TM97; ^#^*P* < 0.05, ^##^*P* < 0.01, ^####^*P* < 0.0001 vs TM98 control. Plasma concentrations below the limit of quantification (LOQ) were calculated as LOQ/2.

The correlation between tumor volume and urinary diacetylspermine that we detected suggested that diacetylspermine may be directly produced by the implanted TNBC-PDX. Therefore, we hypothesized that increased urinary diacetylspermine levels could be a result of tumor SAT1 induction. Plasma diacetylspermine was significantly increased in doxorubicin-treated TM98 when compared to TM97. Yet, plasma levels were generally low and near our limit of quantification (LOQ, Fig. 2a). Tumor levels of other spermine/spermidine-related metabolites like N1-acetylspermidine, N-acetylspermine and diacetylspermine, as well as SAT1 expression, were significantly elevated in TM98 compared with TM97 mice (Fig. 2d, e). Notably, the levels of these metabolites were further increased by doxorubicin treatment in the TM98 doxorubicin responder mice, but not in TM97 mice.

### Increased SAT1 expression and function by doxorubicin is time and dose-dependent

To confirm whether doxorubicin directly induced SAT1, we treated TNBC cell lines with physiologically relevant levels of doxorubicin (1 μM) over a time course. Significant induction of SAT1 by doxorubicin did not occur until 24 h after treatment, which was consistent across cancer cell lines derived from various tumor types (Supplementary Fig. 1).

TM98 mice given doxorubicin continued to produce elevated urine diacetylspermine and upregulated SAT1 expression when compared to control mice at one week after the final dose. To further characterize the pharmacodynamic response, mice given doxorubicin were placed into metabolic cages weekly from day 14 to 35 (Fig. 2f). When normalized to tumor volume, the increase in urine diacetylspermine was sustained for 14 days following the final dose. Therefore, doxorubicin caused a persistent increase in diacetylspermine production long after the drug was cleared (Fig. 2g).

Next, we determined if doxorubicin affected the level of SAT1 induction in a dose-dependent manner. Doxorubicin dose-dependently increased SAT1 expression similarly in TNBC cells and TM98 ex vivo tissue slices with the most robust increase occurring at 1 μM (Supplementary Fig. 2a). Furthermore, elevated acetylated polyamine levels coincided with the increase in SAT1 expression (Supplementary Fig. 2b). Together, these results indicate that elevated urinary diacetylspermine is the result of increased SAT1 expression.

### TNBC ex vivo slices confirm elevated production of diacetylspermine by doxorubicin treatment and recapitulate in vivo tumor SAT1 induction

**Fig. 3:**
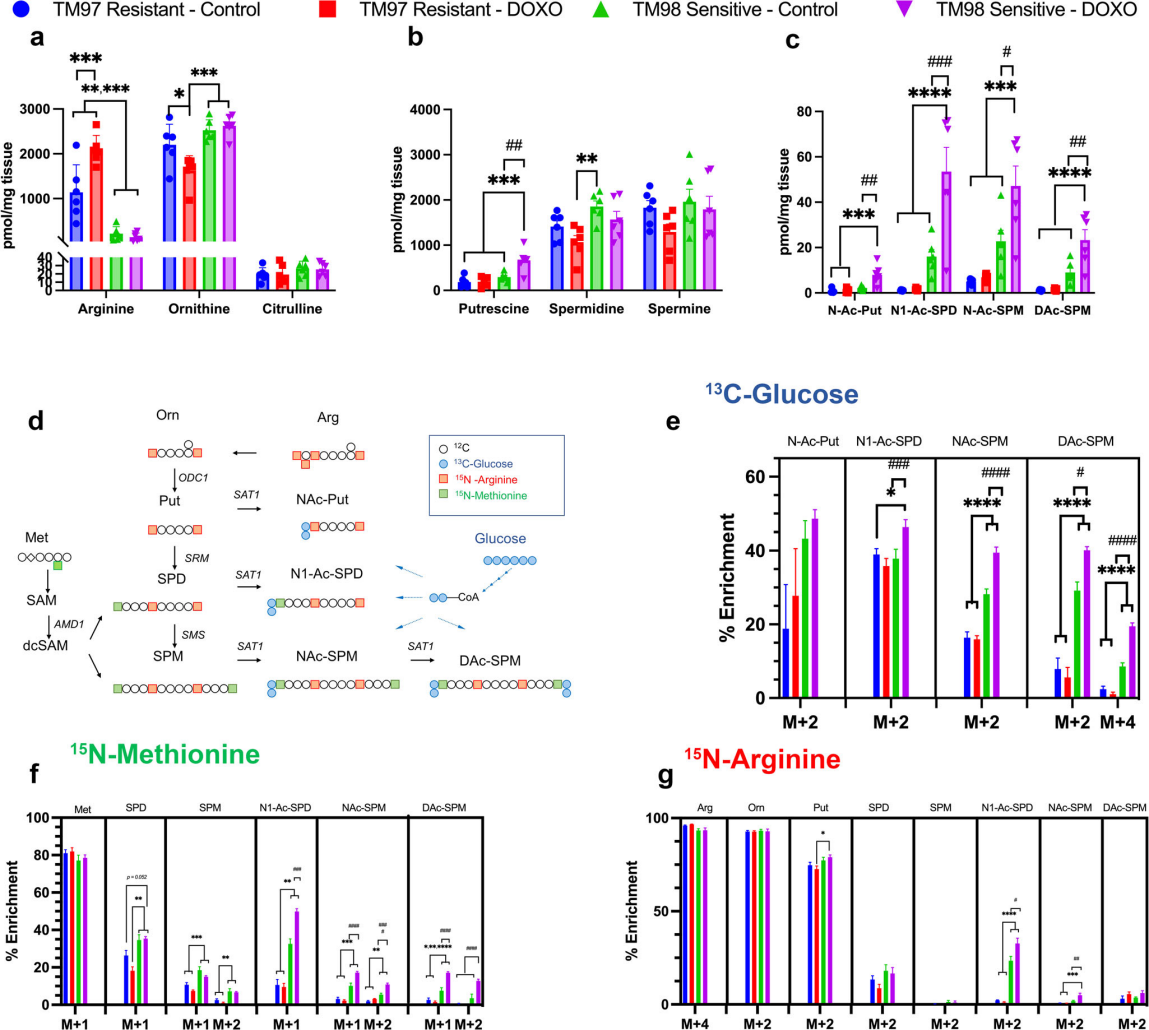
S-Adenosylmethioninamine and acetylated polyamine flux contribute to diacetylspermine production in ex vivo TNBC-PDX tissue slices. Quantified urea cycle (**a**), polyamine (**b**) and acetylated polyamine (**c**) metabolites. **d** Atom transition map of ^13^C-glucose (**e**), ^15^N-methionine (**f**), and ^15^N-arginine (**g**), stable isotope metabolic flux in TM97 and TM98 ex vivo tissue slices treated with vehicle (DMSO) or 1 µM doxorubicin for 24 h. Experiments were performed in triplicate. Data are presented as mean ± s.e.m., *n* = 6. Significance was determined by two-sided one-way ANOVA with Tukey’s test, *P < 0.05, **P < 0.01, ***P < 0.001, ****P < 0.0001 vs TM97; ^#^*P* < 0.05, ^##^*P* < 0.01, ^###^*P* < 0.001, ^####^*P* < 0.0001 vs TM98 control.

To further characterize the effect of doxorubicin on the polyamine pathway, we incubated TM97 and TM98 ex vivo tissue slices with doxorubicin, or vehicle control. After 24-h doxorubicin treatment, acetylated polyamines were significantly increased in TM98 compared to control or compared to TM97 ex vivo slices (Fig. 3c). This occurred without affecting tissue slice viability (Supplementary Fig. 2c). Although metabolites were measured 1 and 7 days after ex vivo slice and in vivo treatment, respectively, ex vivo slices largely captured the in vivo urea cycle and polyamine metabolic profile (Fig. 2b–d and Fig. 3a–c). One noted difference was the increase in putrescine following 24-h doxorubicin treatment suggesting an early onset response to polyamine depletion, which may dissipate in the days following in vivo treatment (Figs. 2c and 3b).

### Polyamine flux contributes to TNBC diacetylspermine production

Besides SAT1, the rate-limiting polyamine anabolic enzymes, ornithine decarboxylase 1 (ODC1) and adenosylmethionine decarboxylase 1 (AMD1), were also increased in TM98 compared to TM97 (Fig. 2e), however, polyamines levels were largely unaltered (Fig. 2c). Therefore, we hypothesized that increased diacetylspermine tumor production is the result of increased metabolic flux through the urea cycle and polyamine pathways. To delineate these pathways, we incubated TM97 and TM98 ex vivo tissue slices with media containing pathway-selective stable isotope-labeled substrates. TM98 ex vivo slices cultured in the presence of ^13^C-glucose showed a significantly elevated incorporation of the ^13^C-label into acetylated spermine metabolites compared to TM97. Moreover, the magnitude of increase was greatest for diacetylspermine (Fig. 3e). The postulated increased polyamine flux in TM98, when compared to TM97, was further verified by ^15^N-methionine enrichment of spermidine and spermine (Fig. 3f), while total metabolite levels remained mostly unaltered (Fig. 3b). A substantially larger fraction of N1-acetylspermidine and N-acetylspermine were +2 labeled suggesting elevated flux of newly generated polyamines towards acetylation in TM98 (Fig. 3f). This flux was further increased by doxorubicin treatment. During ^15^N-arginine treatment, labeled putrescine accumulated, and the stable isotope incorporation pattern was similar to other isotopes for N1-acetylspermidine and N-acetylspermine (Fig. 3g). However, minimal label incorporation occurred in spermidine and spermine.

Polyamines are highly regulated and, when reaching high levels, synthesis is quickly reduced while catabolism increases. To test whether the polyamine pathway was capable of responding to elevated polyamine levels in TNBC-PDX, we treated TNBC-PDX ex vivo slices with vehicle or doxorubicin in the presence of spermine. Spermine treatment dose-dependently decreased putrescine and acetylated polyamines levels in control and doxorubicin treated TM98 ex vivo tissue slices (Supplementary Fig. 3). Spermidine and spermine levels remained unaffected but, at high spermine concentrations, polyamine regulation failed leading to increased diacetylspermine in TM97 and TM98, thus confirming that SAT1 can respond to elevated polyamines to generate diacetylspermine in both tumors (Supplementary Fig. 3g).

### Doxorubicin decreases urinary diacetylspermine in TNBC with high baseline SAT1 function

**Fig. 4:**
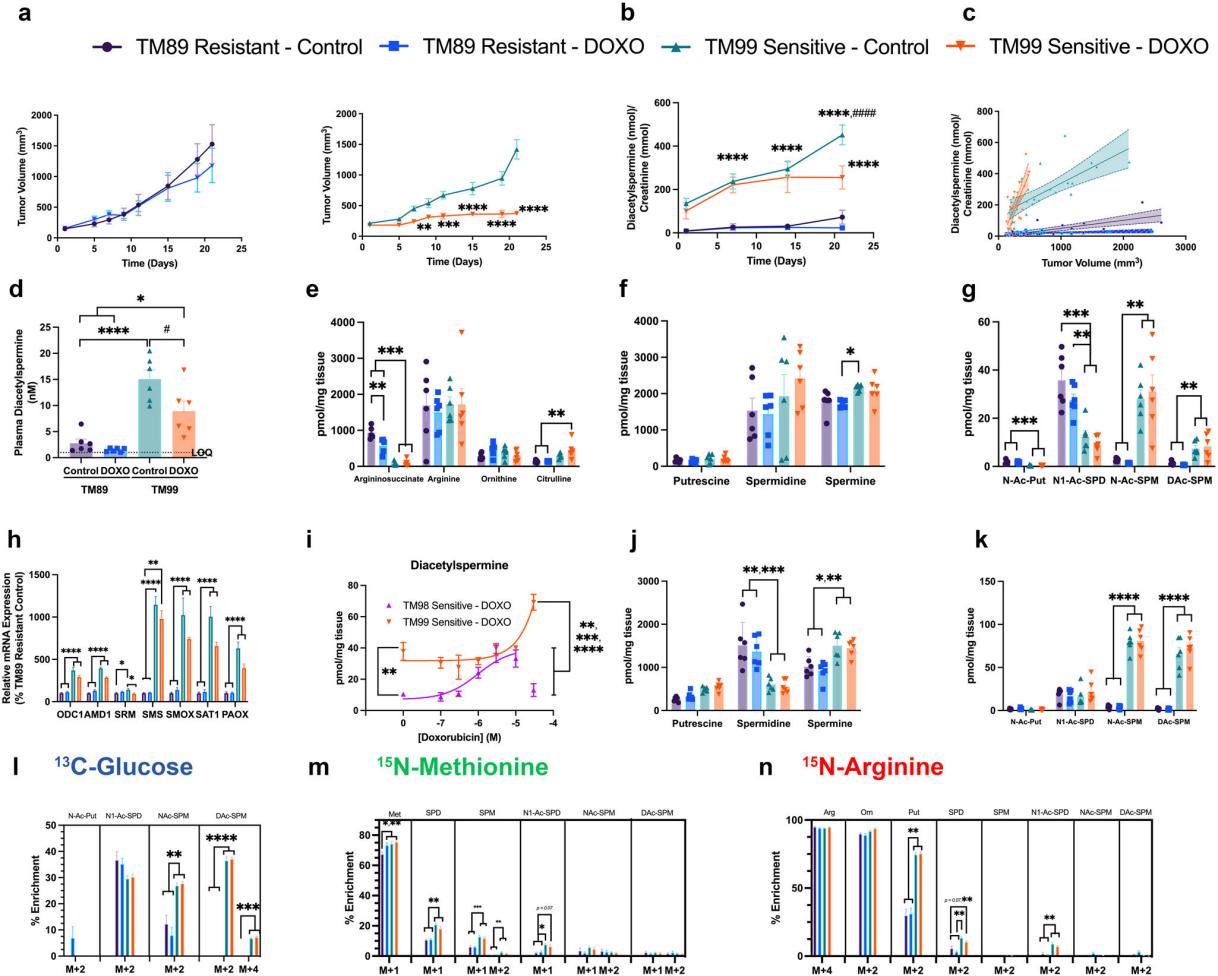
Effective doxorubicin treatment decreases urinary diacetylspermine in sensitive TNBC-PDX with high baseline SAT1 function. **a** Tumor growth curves showing TM89 resistance and TM99 sensitivity after treatment with vehicle (5% dextrose, IV) or doxorubicin (DOXO, 2 mg/kg, IV), mean ± s.e.m., *n* = 6. **b** Quantified urinary diacetylspermine and **c** correlation with tumor volume. **d** Plasma diacetylspermine and tumor urea cycle (**e**), polyamine (**f**) and acetylated polyamine (**g**) metabolites from TM89 and TM99 TNBC-PDX mice, *n* = 6. **h** Doxorubicin dose-response in TM99 and TM98 ex vivo TNBC-PDX tissue slices, *n* = 3. Urea cycle (**i**), polyamine (**j**) and acetylated polyamine (**k**) metabolites demonstrating elevated spermine and spermine acetylation in TM99 compared to TM89 ex vivo tissue slices, independent of doxorubicin (1 µM) treatment. Stable isotope metabolic flux in TM89 and TM99 ex vivo tissue slices treated with ^13^C-glucose (**l**), ^15^N-methionine (**m**), and ^15^N-arginine (**n**), vehicle (DMSO) or 1 µM doxorubicin for 24 h. Ex vivo slice experiments were performed in triplicate, *n* = 6. All data are presented as mean ± s.e.m. Significance was determined by two-sided one-way ANOVA with Tukey’s test or two-way repeated measures ANOVA with Sidak’s correction, *P < 0.05, **P < 0.01, ***P < 0.01, ****P < 0.0001; ^#^*P* < 0.05, ^####^*P* < 0.0001 vs TM99 control.

To further validate that diacetylspermine is a metabolic biomarker of doxorubicin drug response, we used two additional PDX models and treated TM89 (doxorubicin-resistant) and TM99 (doxorubicin-sensitive) TNBC-PDX mice with doxorubicin or vehicle. Tumor growth rates were similar between TM89 and TM99 (Fig. 4a). Urine diacetylspermine levels were increased in TM99 compared to TM89, which correlated with tumor volume and changed similarly in plasma, tumor tissue, and ex vivo tumor slices (Fig. 4).

Interestingly, TM99 responded to doxorubicin treatment through a reduction in tumor size, but tumor diacetylspermine production was unaffected. Measuring *SAT1* mRNA revealed a striking 10-fold greater abundance in TM99 PDX expression compared to TM89 PDX, suggesting a high baseline level of SAT1 in TM99 (Fig. 4h). Furthermore, in TM99 ex vivo tumor slices, baseline diacetylspermine levels were 3.7-fold higher than in TM98 (Fig. 4i). When TM99 ex vivo slices were treated with doxorubicin, a concentration above physiological relevance was necessary to increase diacetylspermine beyond the already elevated baseline production. Therefore, TM99 tumor production of diacetylspermine was unaffected by doxorubicin in vivo but the resulting urine levels predictably decreased in proportion to decreasing tumor size. Indeed urine, plasma, and tumor diacetylspermine significantly correlated across all TNBC-PDX models (Supplementary Fig. 4).

In TM99, in vivo tumor tissue and ex vivo slices demonstrated elevated N-acetylspermine and diacetylspermine levels compared to TM89, but N1-acetyspermidine was either unaffected or decreased, respectively (Fig. 4g, k). Unlike TM98, acetylated spermine metabolites were observed as the major catabolic product of high SAT1 baseline function in TM99 (Fig. 4k and Supplementary Fig. 5). This observation was further supported by increased ^13^C-enrichment of N-acetylspermine and diacetylspermine, but not N-acetylspermidine in TM99 (Fig. 4l). Consistent with the TM98-doxorubicin sensitive tumor, ^15^N-derived from methionine demonstrated increased polyamine flux in TM99 compared to TM89 (Fig. 4m). However, downstream ^15^N-methionine enrichment into acetylated spermine metabolites was undetected in TM99 ex vivo slices. Together these data suggest that in tumors with high baseline SAT1 function, spermine acetylation is the major route of SAT1 metabolism and elevated polyamine flux may not contribute to acetylated metabolites.

### Diacetylspermine is elevated in breast cancer tumors

**Fig. 5:**
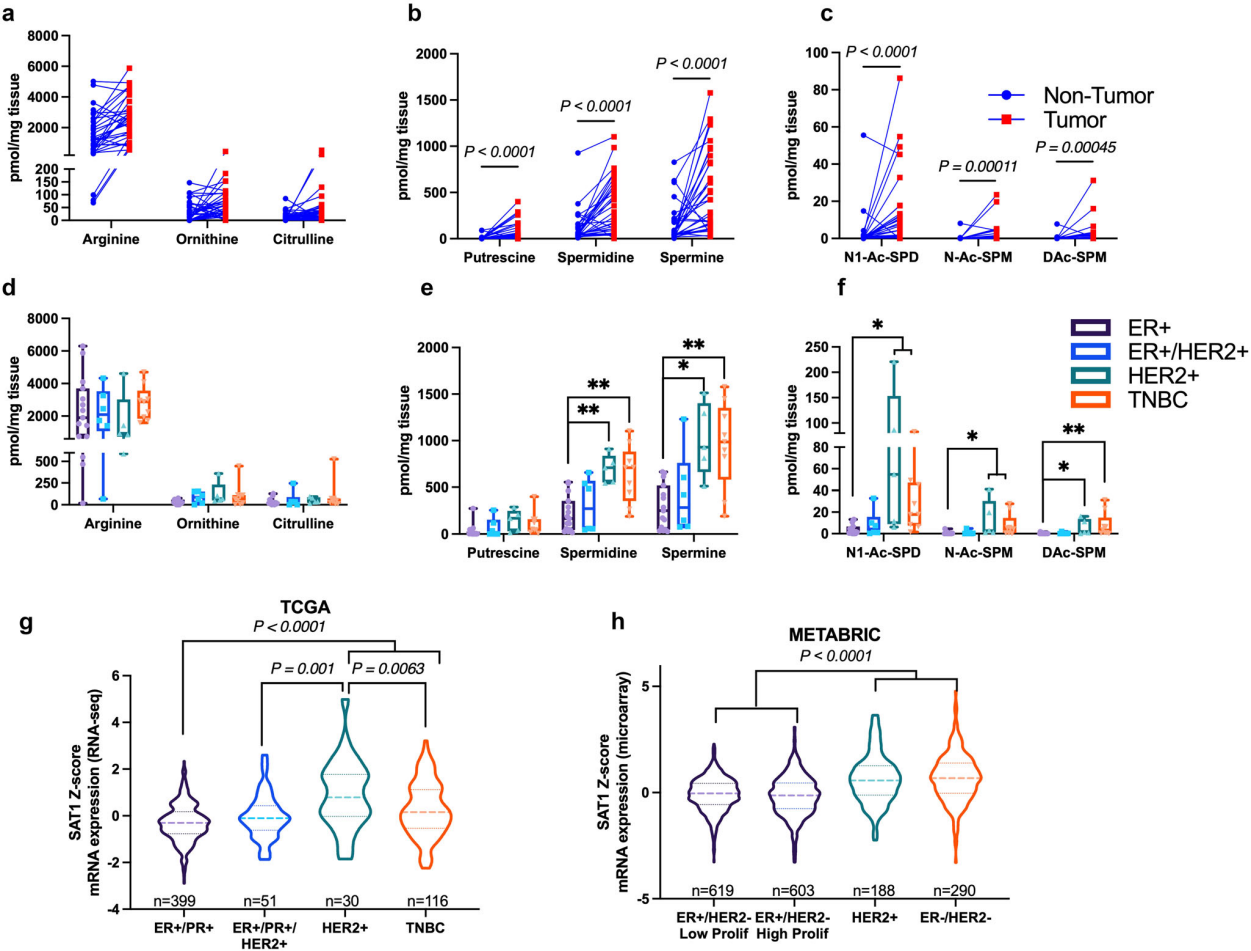
Diacetylspermine is elevated in breast cancer tissue, specifically in TNBC and HER2+ molecular subtypes. Breast cancer tumor vs matched non-tumor tissue levels of urea cycle (**a**), polyamine (**b**) and acetylated polyamine metabolites (**c**), *n* = 33. Urea cycle (**d**), polyamine (**e**) and acetylated polyamine (**f**) metabolites in breast cancer according to molecular subtype classification. **g** SAT1 mRNA expression in TCGA and **h** METABRIC studies were obtained from cBioPortal. Box and swarm plots represent interquartile range (IQR) with center median and 1.5× IQR whiskers. Similarly, violin plots contain center median (dashed line) and 1.5× IQR (dotted lines). Gene expression data are presented in violin plots with *n* values indicated in the figure. Significance was determined by two-sided Wilcoxon matched-pair or one-way ANOVA with Tukey’s test, *P < 0.05, **P < 0.01, ***P < 0.01, ****P < 0.0001.

Our studies in TNBC-PDX indicated that increased urine diacetylspermine is directly produced by the TNBC tumor. Therefore, to determine if diacetylspermine is altered in clinical breast cancer tumors, we measured urea cycle, polyamines, and acetylated polyamines in breast cancer tumor samples and matched non-tumor tissue. Polyamines and acetylated polyamines, including diacetylspermine, were significantly increased in tumor tissue compared to matched non-tumors (Fig. 5b, c). When evaluating breast cancer tumors by molecular subtype, HER2+ and TNBC tumors produced significantly higher levels of spermidine, spermine and acetylated polyamines compared to ER+ tumors (Fig. 5e, f). Moreover, gene expression data from The Cancer Genome Atlas and METABRIC studies demonstrate that SAT1 expression is significantly increased in HER2+ and TNBC tumors compared to ER+ (Fig. 5g, h).

### Urine diacetylspermine increases with tumor size and is elevated in patients with high-grade tumors

**Fig. 6:**
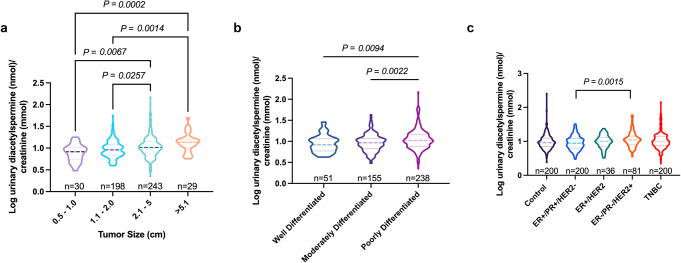
Urine diacetylspermine levels increase with tumor volume and are elevated in patients with poorly differentiated tumors. 12-hour urine diacetylspermine levels relative to tumor volume (**a**), tumor grade (**b**) and molecular subtype (**c**) in breast cancer patients from the Polish Breast Cancer Study (NCT00341458). Diacetylspermine concentrations are log transformed and normalized to creatinine. Data are presented as violin plots with center median (dashed lines) and 1.5× IQR (dotted lines); *n* values indicated in the figure. Significance was determined by two-sided one-way ANOVA with Tukey’s test, *P < 0.05, **P < 0.01, ***P < 0.01.

Given that urine diacetylspermine correlated with TNBC-PDX tumor volume, we next asked whether urine levels were also associated with elevated tumor size in breast cancer patients from a large clinical study. A subset of 12-h urine samples from the Polish Breast Cancer Study (*n* = 717) containing control (*n* = 200), ER+/PR+ (*n* = 200), ER+/HER2 (*n* = 36), ER−/PR−/HER2+ (*n* = 81), and TNBC (*n* = 200) samples, including 500 breast cancer patients with tumor volume information, were analyzed (NCT00341458). Remarkably, urine diacetylspermine levels progressively increased with increasing tumor size in breast cancer patients (Fig. 6a). In addition, urine diacetylspermine was significantly increased in patients with poorly differentiated tumors compared to lower grades (Fig. 6b). However, there was no difference between urine diacetylspermine levels in control subjects and breast cancer patients (Fig. 6c).

## Discussion

Tumor-derived metabolites excreted into blood and urine can be sampled non-invasively to provide an indication of treatment status. Moreover, when these metabolites are also directly altered by the treatment, they can act as an early signal of treatment effectiveness. Here, we conducted a highly controlled pharmacometabolomics study using PDX models and identified diacetylspermine as a metabolic biomarker of doxorubicin effectiveness in TNBC. We then performed a rigorous analysis to characterize tumor diacetylspermine production and determined its potential utility to assess tumor size by measuring urine levels in breast cancer patients.

Our aim was to design a preclinical study that would optimize biomarker identification and remain relevant to clinical sample collection. Therefore, longitudinal urine was collected from TNBC-PDX mice at the time of drug treatment^8^. Repeated-measures analysis resulted in the identification of diacetylspermine as the most significantly altered metabolic feature in our urine metabolomics analysis. Diacetylspermine was previously identified in breast, colon and lung cancers^9–11^, but the effects of drug treatment on urine diacetylspermine and the source of its production have not been directly investigated.

Urine diacetylspermine clearly demonstrated a longitudinal correlation consistent with tumor growth in the TM98 and TM99 doxorubicin-sensitive TNBC-PDX models. However, when TM98 mice were treated with doxorubicin and tumor growth was attenuated compared to vehicle-treated mice, we found a further increase in urine diacetylspermine. Tumor measurements, ex vivo slices and in vitro experiments determined that SAT1 expression was directly induced by doxorubicin, thereby resulting in elevated urine diacetylspermine. This effect is consistent with data from the NCI-60 cell lines treated with doxorubicin. Although stromal cells can play a critical role in doxorubicin drug treatment response^12^, dose-response curves were superimposable for breast cancer cell lines and TM98 ex vivo slices suggesting that cancer cells rather than stromal cells are responsible for diacetylspermine secretion.

We collected urine from mice 7 days after the final doxorubicin dose and, in doing so, we discovered that urine diacetylspermine levels continued to increase long after doxorubicin had been systemically cleared. Indeed, the effects of doxorubicin on gene expression were shown to outlast its elimination^13^. Upon further study, we demonstrated that elevated SAT1 occurs 24 h after treatment and that diacetylspermine production lasts for 14 days following the final doxorubicin dose. These data suggest a clinically amenable timeframe for measuring diacetylspermine production as an indicator of doxorubicin effectiveness in TNBC. Although elevated urine diacetylspermine does not distinguish between increased tumor growth and doxorubicin-induced diacetylspermine tumor production in the TM98 TNBC-PDX model, patient tumors grow at a much slower rate than PDX tumors and therefore, elevated urine diacetylspermine in patients early during treatment would be mainly indicative of doxorubicin effectiveness.

Cancer cells require polyamines for growth and proliferation. To meet this demand, altered signaling pathways during cancer reprogramming upregulate the rate limiting anabolic enzymes, ODC1 and AMD1^14^. Elevated SAT1 and decreased levels of PAOX were previously demonstrated in breast cancer, creating a favorable environment for diacetylspermine production^15^. SAT1 responds to elevated free polyamines by increasing polyamine acetylation^16^. This mechanism was shown in a breast cancer cell line^17^ and was present when ex vivo slices were treated with spermine regardless of doxorubicin sensitivity or baseline SAT1 expression and diacetylspermine production. A significant advantage of the TNBC-PDX models is the ability to resect tumors and culture ex vivo slices, thereby directly assessing tumor metabolic flux using stable isotope tracers^18,19^. Spermidine and spermine levels were similar between TM98 and TM97 in ex vivo slices; however, ODC1 and AMD1 expression as well as incorporation of ^15^N from methionine and arginine were elevated in TM98, demonstrating increased polyamine flux. Increased tracer levels were also evident in acetylated metabolites. These data suggest that acetylation may be increased to keep polyamine levels under tight control, thereby leading to elevated diacetylspermine. Conversely, high-baseline expression and activity of SAT1 in TM99 did not necessitate elevated polyamine flux for the generation of acetylated polyamines, suggesting an alternate mechanism of increased SAT1 expression at baseline in this model. Furthermore, the preferential generation of acetylated spermine metabolites in TM99 may be indicative of spermine availability^17^.

Doxorubicin-mediated induction of SAT1 demonstrated a pattern similar to previous evidence of overexpression, whereby putrescine levels increase in response to polyamine depletion, further indicating a direct effect on SAT1^20^. A recent study described a doxorubicin-mediated decrease in ODC1 activity 48 h after treatment, which suggests that the increase at 24 h may dissipate^21^. This effect may contribute to reduced tumor growth; however, ODC1 levels were unaltered 7 days after doxorubicin treatment in vivo. In addition, polyamine flux was elevated during SAT1 overexpression in a previous study but, in ex vivo tissue slices, doxorubicin treatment only affected acetylated polyamine flux^20^. Together, these data confirm that doxorubicin treatment mainly affects diacetylspermine levels by modulating SAT1 expression and activity. Further study is necessary to determine whether SAT1 activity contributes to the doxorubicin efficacy.

Although doxorubicin largely induces SAT1 expression at physiological concentrations as demonstrated in TM98 and NCI-60 cell lines, doxorubicin decreased plasma and urine diacetylspermine in TM99, which coincided with decreasing tumor size. However, urine and ex vivo tumor diacetylspermine concentrations as well as ^13^C-glucose tracer incorporation were greater than twofold higher in TM99 than TM98. Our data provide evidence that the mechanism of doxorubicin induction of SAT1 did occur in TM99, but only at supraphysiologic concentrations. We propose that high-baseline levels of SAT1 and diacetylspermine production mask the induction by doxorubicin at physiological levels. Therefore, these data shed light on the potential use of urine diacetylspermine as an indicator of therapeutic effectiveness when the treatment does not directly affect SAT1 expression and function. Under these conditions, effective therapy is predictably indicated by a decrease in urine diacetylspermine.

The utility of diacetylspermine as a therapeutic biomarker depends on elevated tumor production. We compared polyamines and acetylated polyamines in tumor tissue with matched control tissue and found that all polyamines and acetylated polyamines were significantly elevated. Therefore, the majority of breast cancer tumors produce elevated levels. Notably, acetylated polyamines were increased in HER2+ and TNBC tumors when compared to ER+ molecular subtypes, which was consistent with TCGA and METABRIC expression levels of SAT1. Chemotherapy is commonly used to treat TNBC and HER2+ breast cancer^22^ and therefore, these patients are more likely to benefit from therapeutic biomarker monitoring.

Diacetylspermine excreted from tumor tissue is readily cleared into urine creating a reservoir to measure in vivo production^23^. Although several studies have identified diacetylspermine as a cancer biomarker, the current work demonstrates that urine diacetylspermine increases with increasing tumor volume in TNBC-PDX mice and in breast cancer patients. Moreover, elevated urine diacetylspermine was found in patients with poorly differentiated tumors, consistent with previous evidence in colorectal cancer^24^. Others recently demonstrated elevated plasma diacetylspermine in TNBC patients when compared to healthy subjects^11^. In contrast, we did not find significant differences in urine diacetylspermine levels between control and breast cancer patients. This may be explained by interindividual variability. Although diacetylspermine production is elevated in breast cancer tumors, polyamines are ubiquitous and factors influencing polyamine metabolism such as age^25^ and inflammatory diseases^26^ can also affect urine diacetylspermine levels. Our results therefore, highlight the need for the collection of baseline and longitudinal samples in future clinical studies to assess the utility of urine diacetylspermine as a metabolic biomarker of treatment effectiveness. Importantly, these data suggest that breast cancer tumor size is associated with urine diacetylspermine, which is a fundamental feature for a biomarker of therapeutic effectiveness. Evaluating urine diacetylspermine during treatment may provide a surrogate indicator of tumor size in real-time through routine urine collection and without the need for repeated imaging or histological evaluation. Moreover, early identification of doxorubicin resistance may lead to termination of ineffective treatment, reducing unnecessary anthracycline-based therapy and avoiding exposure that can increase the risk of long-term cardiotoxicity. To this end, our data suggest that effective doxorubicin treatment may ultimately lead to reduced urine diacetylspermine levels in patients. In addition, doxorubicin induction of SAT1 may initially increase urine diacetylspermine levels in indicating that a patient will respond to treatment, prior to the decrease in tumor volume, as an early marker of treatment effectiveness.

There are several limitations to our study. Although we identified doxorubicin-sensitive TNBC-PDX models with elevated diacetylspermine production, consistent with human tumors, all doxorubicin-resistant models did not produce significant levels of diacetylspermine. Future studies are necessary to determine whether low diacetylspermine is a characteristic of doxorubicin resistance. Breast cancer tumor and urine samples were not obtained from the same study and therefore, we are unable to directly compare tumor and urine levels in patients. Only a single treatment naive urine sample was obtained from breast cancer patients, therefore longitudinal sampling and response to doxorubicin treatment remains to be evaluated clinically.

Our study has several strengths. First, all TNBC-PDXs consistently demonstrated sensitivity or resistance to doxorubicin over multiple passages throughout the study, confirming the robustness of these models. Second, TNBC tumor variability was captured by using multiple TNBC-PDX models to provide a broad picture of diacetylspermine production and response to doxorubicin treatment. Third, culturing ex vivo tumor slices allowed us to assess polyamine metabolism and metabolic flux directly from our in vivo models. Finally, we identified an association between tumor volume and urine diacetylspermine in our animal studies and similarly in a large breast cancer case-control study using urine collected for 24 and 12 h, respectively. This approach mitigated any behavioral differences between mice and humans when voiding urine, and provided a measure of steady-state urine diacetylspermine levels.

Using a pharmacometabolomics approach, we discovered that urine diacetylspermine is a metabolic biomarker of doxorubicin effectiveness in TNBC-PDX models and is associated with tumor volume. Our findings provide evidence that urinary diacetylspermine may be a clinical biomarker of breast cancer treatment effectiveness. Moreover, this approach can be applied to other cancer types and drug treatment regimens. Future prospective clinical studies are necessary to determine the utility of urinary diacetylspermine as a guide to precision therapy.

## Methods

### Mouse models and cell lines

TNBC patient-derived xenograft models TM00089 (TM89 doxorubicin-resistant, BR0620F), TM00097 (TM97 doxorubicin-resistant, BR1077F), TM00098 (TM98 doxorubicin-sensitive, BR1126F) and TM00099 (TM99 doxorubicin-sensitive, BR1367F) were obtained from Jackson Laboratories between passages 3 and 7. All animal studies were approved by the NCI Institutional Animal Care and Use Committee (IACUC). NSG mice were obtained for NCI Frederick or Jackson Laboratories. BT549 and HS578T cells were obtained from the NCI Developmental Therapeutics Program (DTP) repository.

### TNBC-PDX in vivo studies

TNBC-PDX tumors were surgically resected from TNBC-PDX mice, cut into 2 × 2 × 2 mm and subcutaneously implanted into 5- to 8-week-old female mice. When tumors reached 100–200 mm^3^, TNBC-PDX mice were randomized and placed into control or doxorubicin groups. Subsequently, mice were given doxorubicin (2 mg/kg, Cayman Chemicals) or vehicle (5% dextrose) by intravenous injection into the lateral tail vein once weekly for 3 weeks. Tumors were measured thrice weekly using calipers and tumor volumes were calculated using the following formula: volume = (length × width^2^)/2. For urine collection, mice were placed in autoclaved glass metabolic cages (Jencons, Metabowl) and contained in a HEPA-filtered laminar flow hood for 24 h. TNBC-PDX mice were placed in metabolic cages on days 1, 7, and 14, immediately following each drug administration. At 21 days, a final 24-h urine collection was performed in the absence of drug treatment followed by euthanization to collect plasma and tissue, unless otherwise indicated. Urine samples were centrifuged at 2000 g and the supernatant was frozen at −80 °C.

### Ex vivo tissue slice experiments

Untreated TNBC-PDX mice with tumors between 1000 and 1500 mm^3^ were euthanized and tumors excised under sterile conditions. Tumors were sliced once along the major-axis to create a flat surface and covered with 2% low melting-point agarose heated to ~40 °C^19^. When agarose was hardened, tumors were cut into 500 µm slices using a McIlwain Tissue Chopper (Ted Pella, Inc.) and washed twice in PBS. Ex vivo slices were placed in 6-well plates containing 3 mL of media then onto an orbital shaker in a humidified 37 °C incubator under 5% CO_2_. RPMI containing 10% FBS, 100 U/mL penicillin/streptomycin/antimycotic was used to culture ex vivo slices. For isotopic labeling experiments, tumor slices were cultured in RPMI containing 10% dialyzed FBS, 100 U/mL penicillin/streptomycin/antimycotic and unlabeled or uniform labeled ^13^C-glucose (in RPMI cat. 11879020), ^15^N-methionine (in RPMI cat. A1451701) or ^15^N-arginine (in RPMI cat. 88365 plus L-lysine). After 24 h, tissue slices were removed, washed with PBS, blotted dry, weighed and flash frozen in liquid nitrogen. Viability after 24-h treatment was compared to fresh ex vivo slices using PrestoBlue, following the manufacture's protocol (cat. A13261)^19^.

### Untargeted urine metabolomics analysis

Urine creatinine concentrations were measured using the Jaffe method. To reduce urine concentration variability for metabolomics analysis, all samples were diluted to one standard deviation below the mean creatinine concentration. Samples were then diluted 1:5 in acetonitrile/water/methanol (65/30/5) containing 10 µM α-aminopimelic acid as internal standard and injected onto a Waters H-Class ultra-performance liquid chromatography (UPLC) coupled to a Waters Xevo G2 quadrupole time of flight MS (QTOFMS) in positive and negative ionization mode, as previously described^27^. Briefly, chromatographic separation was performed using an Acquity BEH amide column (2.1 × 50 mm) maintained at 40 °C and a flow rate of 0.4 mL/min in a Waters H-Class UPLC. Mobile phase consisted of 10 mM ammonium acetate in 90% acetonitrile (A, pH = 9.0) and 10 mM ammonium acetate in 10% acetonitrile (B, pH = 9.0). Accurate mass correction was performed using leucine-enkephalin. Urine metabolomics raw data files were processed using Progenesis QI software and normalized to creatinine. The resulting feature lists for positive and negative mode were combined after filtering features that ionize in both modes using previously published code^27^.

### Polyamine and amino acids targeted LC-MS analysis

Urine and plasma samples were diluted 1:3 with 6% trichloroacetic acid (TCA)^28^ containing internal standards (1 µM 1,7-diaminoheptane, 100 nM d6-diacetlyspermine and 10 µM d8-spermine). Tissue and ex vivo slice samples were diluted with 20 µL/mg of tissue in 6% TCA containing internal standards and processed in a Precellys homogenizer (6500 RPM for 30 s × 2). Samples were centrifuged at 15,000 × *g* for 10 min and 10 µL of supernatant was added to 70 µL of 100 mM borate buffer containing 100 µM NaOH (pH = 9), 1 mM ascorbic acid and 1 mM TCEP^29^. Samples were derivatized with 20 µL of 6-aminoquinolyl-N-hydroxysuccinimidyl carbamate (AQC, AccQ-Tag, Waters) followed by a 10-min incubation at room temperature and a 10-min incubation at 55 °C to quench the reaction. The resulting derivatized sample was injected onto a Waters Acquity BEH Phenyl-Hexyl column (2.1 × 50 mm) maintained at 40 °C and a flow rate of 0.6 mL/min in a Waters I-Class UPLC. The mobile phase consisted of water + 0.1% formic acid (A) and acetonitrile + 0.1% formic acid (B) and run under the following conditions: 0–0.5 min, 0 %B; 0.5–4 min, 0–15% B; 4–6 min, 15–30% B; 6–6.5 min, 30–99% B, 7.5–9 mins 0% B. Eluting metabolites were measured in a Waters Xevo TQS using multiple reaction monitoring (MRM, Supplementary Table 2). Parent *m/z* values were calculated using the following equation:

$$[M + (n \times AQC) + (z \times H)]^{z + }$$

where *n* is the number of derivatized amines with *AQC* and *z* is the charge number of the ion. The most sensitive ion charge was used for each metabolite. Spermine was normalized to d8-spermine and all other metabolites were normalized to d6-diacetylspermine or diluted 20-fold and normalized to 1,7-diaminoheptane.

### Stable isotope metabolic flux analysis

Ex vivo slices incubated with media containing stable isotope-labeled compounds were processed as described above and measured using a Waters Synapt G2-S operating in positive mode with a 0.5 kV and 40 V, capillary and cone voltage, respectively. The source temperature was set to 150 °C and the desolvation gas flow rate was 950 L/h at 500 °C. Ions generated from isotopic enrichment of ^13^C or ^15^N were monitored for amino acids and polyamines as described in Supplementary Table 2. IsoCorrectoR was used to correct for natural isotope abundance^30^.

### Human-specific primer design and qPCR

Total RNA was extracted using Trizol and cDNA synthesized with qScript cDNA Supermix, following the manufacturers' protocols. Relative mRNAs levels were quantified with PerfeCTa SYBR Green (Quantabio) and gene expression normalize to human *GAPDH* using the delta-delta *C*_*T*_ method. Human-specific primers were designed in non-overlapping regions of mouse and human transcript orthologs (Supplementary Table 3). Human primer specificity was validated by qPCR using cDNA generated from BT-549 human breast cancer cells and mouse liver tissue.

### Human tissue and urine specimens

Fresh-frozen tumor samples were obtained from unselected breast cancer patients at all disease stages having a mastectomy between February 15, 1993, and August 27, 2003, under the NCI resource contract “Collection and Evaluation of Human Tissues and Cells from Donors with an Epidemiology Profile”, as previously described^31^. The collection of tissue was approved by the University of Maryland Institutional Review Board for participating institutions. The research was also reviewed and approved by the NIH Office of Human Subjects Research Protections. All patients provided written informed consent.

Urine samples were obtained from the Polish Breast Cancer Study, which comprised of a population-based breast cancer case-control study in Poland between 2000 and 2003, as previously described^32^ (NCT00341458). All participants provided written informed consent to participate in the study in accordance with the National Cancer Institute and local Institutional Review Boards. Urine samples were randomly selected from the 2241 control and 1962 breast cancer study subjects as follows: 200 control, ER+/PR+, and TNBC as well as all ER+/PR+/HER2+ (*n* = 36) and HER2+ (*n* = 81).

### Statistical analysis

Principal component analysis was performed on resulting features in SIMCA (version 15). For univariate statistical analysis, features were analyzed by repeated measure two-way ANOVA using Metaboanalyst^33^. *P* values were adjusted according to the Benjamini Hochberg procedure and *q* < 0.05 was considered significantly different. All other statistical analysis methods were performed as indicated in figure legends using GraphPad Prism version 8 and 9.

### Reporting summary

Further information on research design is available in the Nature Research Reporting Summary linked to this article.

## Supplementary information

Supplemental Material

Supplemental Data 1

REPORTING SUMMARY

## Data Availability

Data were downloaded from The NCI Transcriptional Pharmacodynamics Workbench were downloaded from GSE116436^34^. SAT1 expression data from METABRIC and TCGA were downloaded from cBioPortal^35,36^. Raw metabolomics data is available from MetaboLights ID:MTBLS5764. Other data underlying this article will be shared on reasonable request to the corresponding authors.

## Code availability

Metabolomics data was analyzed using Progenesis QI. Positive and negative ionization mode feature lists were combined using previously published code^27^. Statistical analysis was performed using SIMCA 15 and Metaboanalyst 3.0. The custom in-house code used to transform data into the correct format for each program is available from the corresponding authors upon reasonable request.
