# Supplementary material for: Pharmacometabolomics reveals urinary diacetylspermine as a biomarker of doxorubicin effectiveness in triple negative breast cancer
Source: NPJ Precis Oncol. 2022 Oct 7;6:70. doi: 10.1038/s41698-022-00313-4 (PMC9547066; doi:10.1038/s41698-022-00313-4)
Supplement: Supplementary file 1 — Supplemental Material [file 41698_2022_313_MOESM1_ESM.pdf]

Supplementary Figures

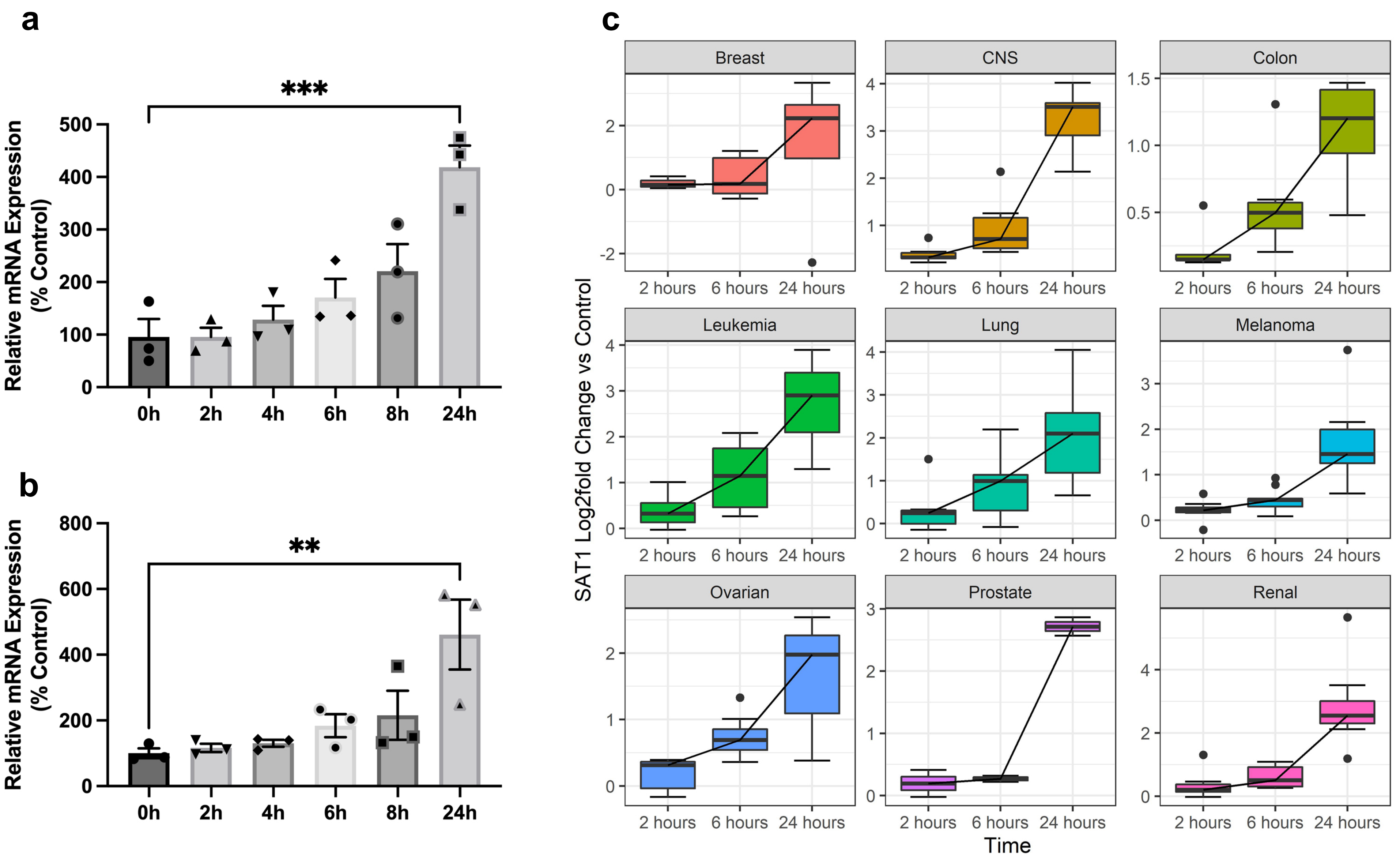

Supplementary Fig. 1 SAT1 induction by doxorubicin is time-dependent. SAT1 mRNA expression after 1 µM doxorubicin treatment at the indicated time points in BT549 (**a**), HS578T (**b**) and NCI-60 cells lines (**c**). Data are presented as mean s.e.m , n = 3 (a,b), or were downloaded from GSE116436 and are presented as box and whisker plots of SAT1 log_2-fold change compared to control at the same timepoint (c)[35]. Significance was determined by one-way ANOVA with Tukey's test.

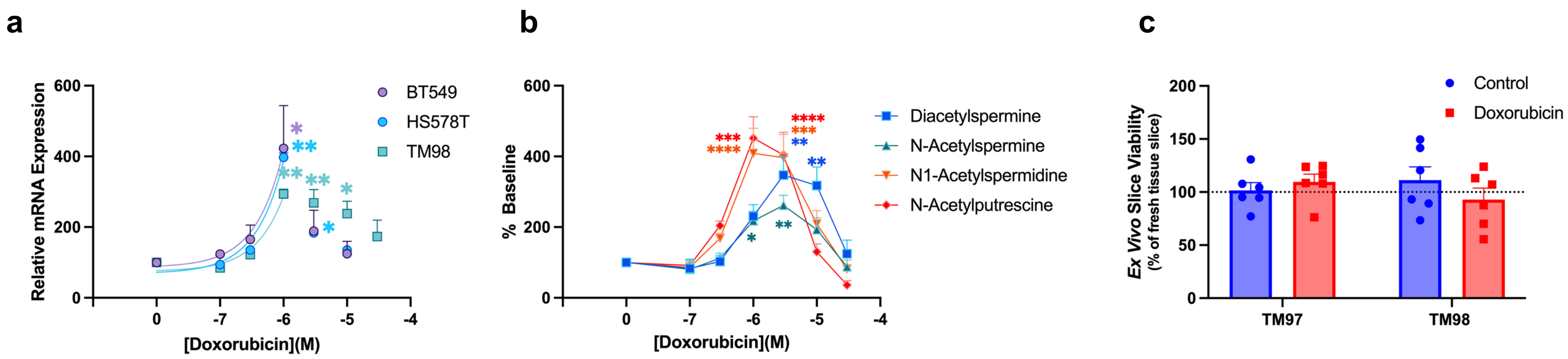

Supplementary Fig. 2. Doxorubicin dose-dependently induces SAT1 mRNA expression and polyamine acetylation. BT549, HS578T and TM98 SAT1 mRNA expression (**a**) and polyamine acetylation (**b**) 24 hours after treatment with doxorubicin (n=3). **c**, Tissue viability of TM97 and TM98 ex vivo slices 24 hours after treatment with vehicle or doxorubicin and normalized to fresh ex vivo slices (n=6). Experiments were performed in triplicate. Data are presented as mean ± s.e.m. *P<0.05, **P<0.01, ***P<0.001, ****P<0.0001 compared to control. Significance was determined by one-way ANOVA with Tukey's test.

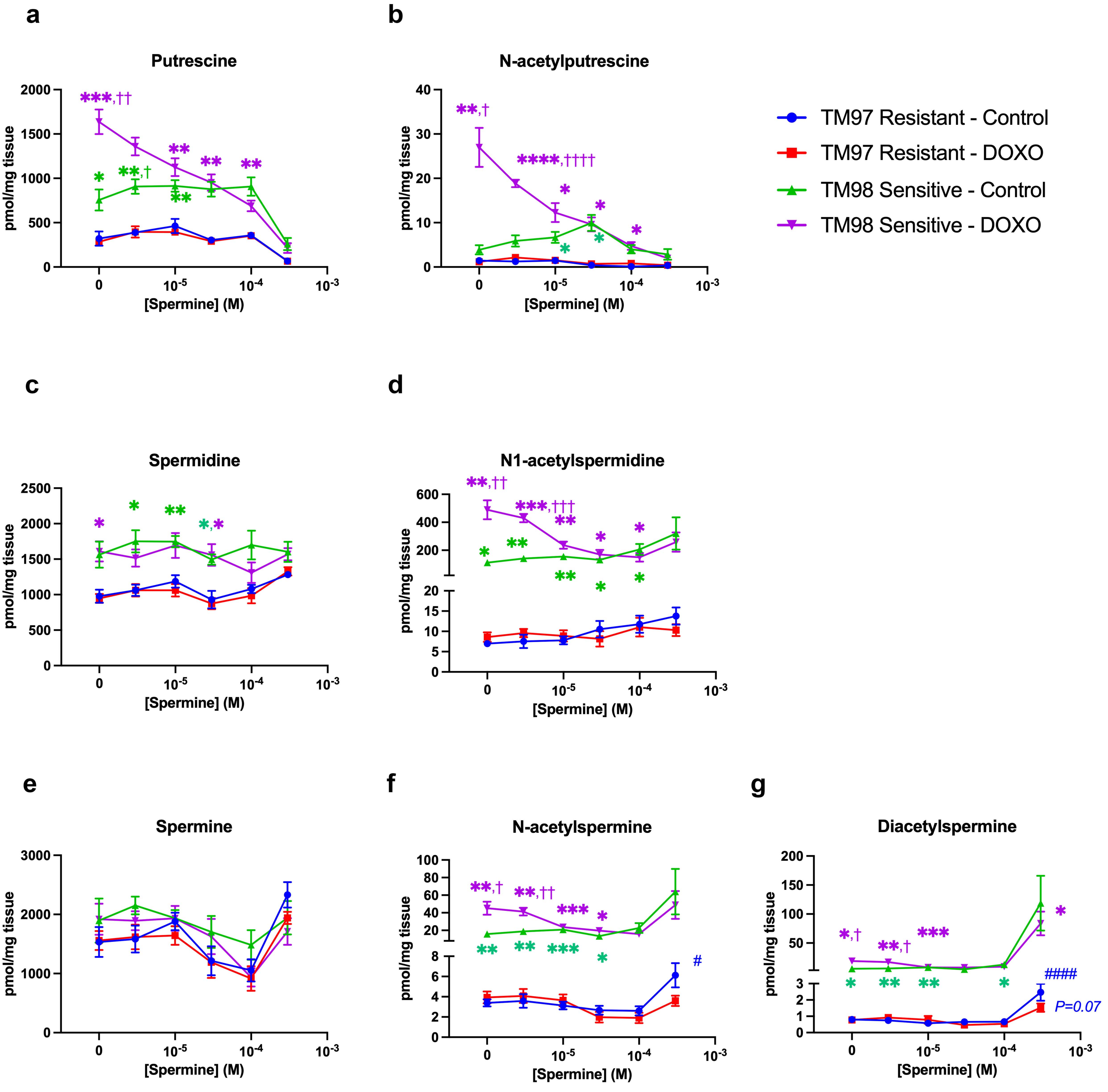

Supplementary Fig. 3. Spermine dose-dependently reverses doxorubicin induced polyamine acetylation in TM98, but at high concentrations, spermine increases diacetylspermine production regardless of doxorubicin sensitivity. Putrescine (**a**), N-acetylputrescine (**b**), spermidine (**c**), N1-acetylspermdine (**d**), spermine (**e**), N-acetylspermine (**f**) and diacetylspermine (**g**) in TM97 and TM98 *ex vivo* tissue slices treated with vehicle or doxorubicin (DOXO) 1 µM in the presence of spermine for 24 hours. Experiments were performed in duplicate and data are presented as mean ± s.e.m, n = 6. **P*<0.05, ***P*<0.01, ****P*<0.001, *****P*<0.0001 vs TM97; $^{\#}$ *P*<0.05, $^{\#\#\#\#}$ *P*<0.0001 vs TM97 in the absence of spermine; †*P*<0.05, ††*P*<0.01, †††*P*<0.001, ††††*P*<0.0001 vs TM98 control. Significance was determined by one-way ANOVA with Tukey's test.

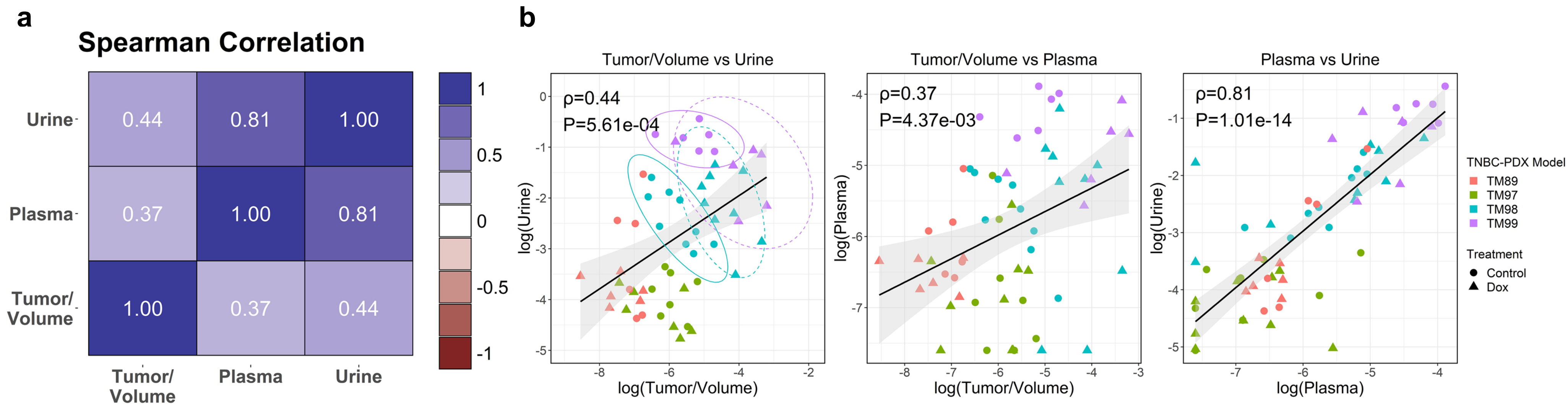

Supplementary Fig. 4. Urine diacetylspermine directly correlates with plasma and tumor levels. (**a**), Spearman rank correlation coefficients between urine, plasma and tumor/volume diacetylspermine levels. (**b**), individual correlation plots show that untreated TM99 produces more urine diacetylspermine per tumor volume than other tumors and that doxorubicin treatment increases TM98 diacetylspermine tumor production, which is reflected in urine. Tumor concentration was normalized to tumor volume and urine diacetylspermine was normalized to creatinine. All data were log transformed; TM97 and TM98 n = 8-9, TM89 and TM99 n = 6. Significance was determined by spearman correlation and P values are annotated.

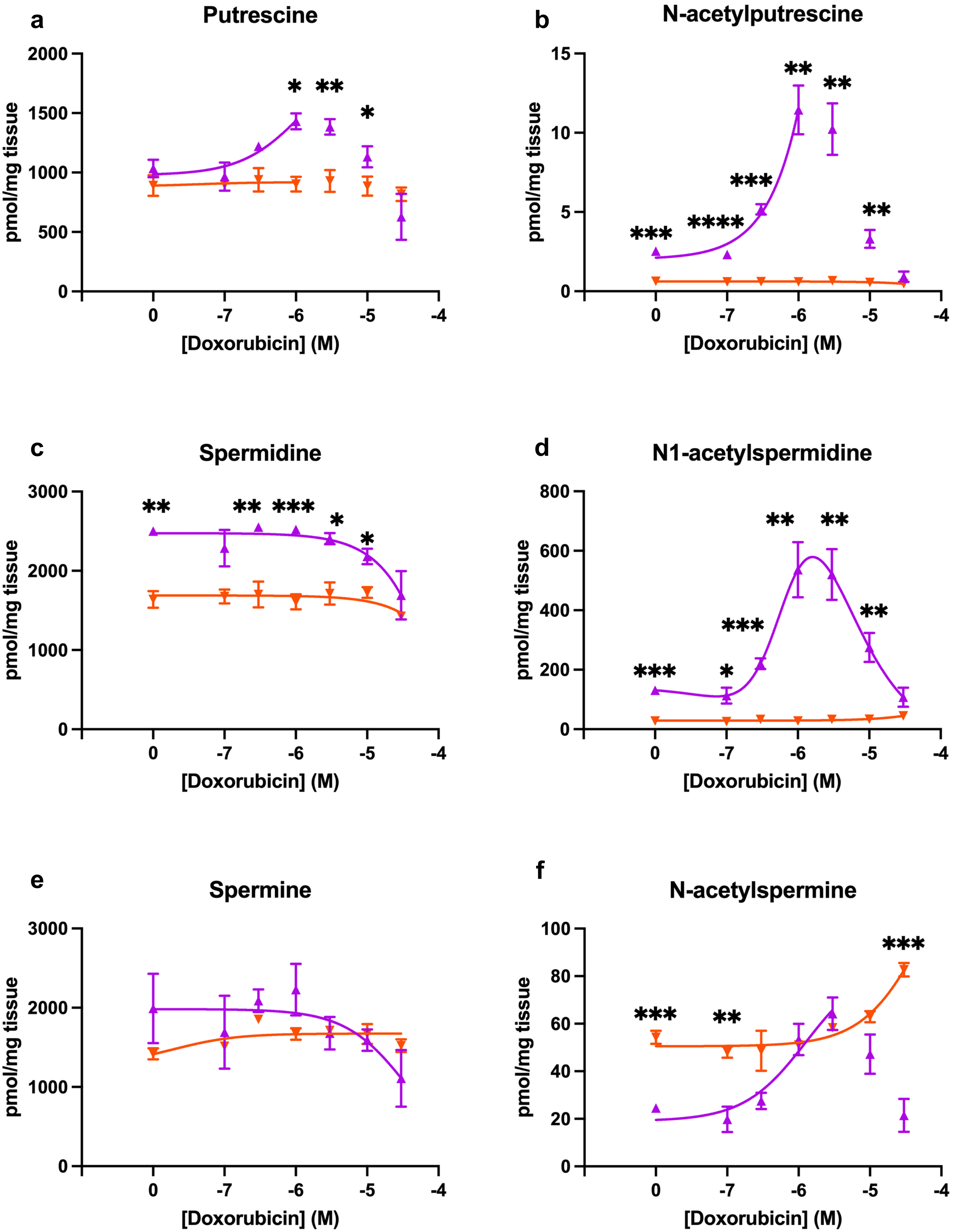

Supplementary Fig. 5. Doxorubicin dose-dependently induces polyamine acetylation in TNBC-PDX with low baseline SAT1 function, but preferentially increases spermine acetylation in TNBC-PDX high baseline SAT1 function. Putrescine (**a**), N-acetylputrescine (**b**) spermidine(**c**), N-acetylspermidine (**d**), spermine (**e**) and N-acetylspermine (**f**) in TM98 (▲) and TM99 (▼) ex vivo tissue slices 24 hours after treatment with doxorubicin. Experiments were performed in triplicate and data are presented as mean ± s.e.m, n = 6. *P<0.05, **P<0.01, ***P<0.001, ****P<0.0001. Significance was determined by t-test. Data are fitted with three parameter or bell-shaped dose response curves.

Supplemental Table 2. List of AQC-derivatized MRM transitions and accurate m/z calculations for neutral and stable-isotope labeled urea cycle and polyamine metabolites

										15N-labelling				13C-labelling			
Number	Name	Elemental Comp	M	n	Aqc(n)	H(n)	(n)+	MRM transition	Accurate m/z	M+1	M+2	M+3	M+4	M+1	M+2	M+3	M+4
1	L-Arginine	C6H14N4O2	174.1117	1	170.048	1.007		345.2 > 70	345.1667	346.1970	347.1941	348.1911	349.1881				
2	L-Argininosuccinic Acid	C10H18N4O6	290.1226	1	170.048	1.007	1	461.2 > 70	461.1776								
3	L-Citrulline	C6H13N3O3	175.0957	1	170.048	1.007	1	346.2 > 145	346.1507								
4	L-Ornithine	C5H12N2O2	132.0899	2	170.048	1.007	2	237.1 > 171	237.1000	237.5985	238.0970	238.5956	239.0941				
5	Putrescine	C4H12N2	88.1515	2	170.048	1.007	2	215.1 > 171	215.1308	215.6293	216.1278	216.6264	217.1249				
6	Spermidine	C7H19N3	145.1579	3	170.048	1.007	2	219.4 > 171	328.6580	329.1565	329.6550	330.1536	330.6521				
7	Spermine	C10H26N4	202.2157	4	170.048	1.007	2	221.6 > 171	442.2109	442.7094	443.2079	443.7065	444.2050				
8	N-Acetylputrescine	C9H21N3O	130.1106	1	170.048	1.007	1	301.2 > 145	301.1656	302.1626	303.1597	304.1567	305.1537	302.1690	303.1723	304.1757	305.1790
9	N1-Acetylspermidine	C9H21N3O	187.1685	2	170.048	1.007	2	264.6 > 100	264.6393	265.1378	265.6363	266.1349	266.6334	265.1410	265.6427	266.1443	266.6460
10	N-Acetylspermine	C12H28N4O	244.2263	3	170.048	1.007	3	252.5 > 171	252.4638	252.7961	253.1285	253.4608	253.7932	252.7983	253.1327	253.4672	253.8016
11	N1,N12-Diacetylspermine	C14H30N4O2	286.2369	2	170.048	1.007	1	314.2 > 100	627.3399	628.3369	629.3340	630.3310	631.3280	628.3433	629.3466	630.3500	631.3533
12	d6-Diacetylspermine	C14H24D6N4O2	292.2745	2	170.048	1.007	1	317.2 > 171	633.3775								
13	d8-Spermine	C10H18D8N4	210.2660	4	170.048	1.007	2	446.2 > 171	446.2360								
14	1,7-Diaminoheptane	C7H18N2	130.1470	2	170.048	1.007	2	236.1 > 171	236.1285								

Supplemental Table 3. List of human specific primers used in this study

Name	Direction	Sequence
SAT1	Forward	ATGTTCAAATGCGCAGCTCTT
	Reverse	GGAGCGTCCTCTTCTCAGTC
GAPDH	Forward	GCTCATTTCCTGGTATGACAACG
	Reverse	GAGATTCAGTGTGGTGGGGG
AMD1	Forward	TGTTTTGTTTTGTTTTGTTTCCAATAG
	Reverse	TCTCTTTTCTAGGCTTTGTCCCA
ODC1	Forward	TGCAGCCATATGGAAGACTAGG
	Reverse	GGCACTCTTGAGTAGCGTGT
SMS	Forward	CGCCTGTATTGTCCTGTGGAA
	Reverse	AGGGGCTACTGATCTTCAGGG
SRM	Forward	GTGTCTGTCTCTCTGGCGTT
	Reverse	TTTGGTGAGTGAGGGGCAAC
SMOX	Forward	CTTCCGCGGCTCCTATTCAT
	Reverse	CTGCATGGGCGCTGTCTTT
PAOX	Forward	CCACGTTCTCTGTGGGTTCA
	Reverse	CGTGGGTTTCCTGTCACTCT
