# Supplementary material for: Pharmacometabolomics reveals urinary diacetylspermine as a biomarker of doxorubicin effectiveness in triple negative breast cancer
Source: NPJ Precis Oncol. 2022 Oct 7;6:70. doi: 10.1038/s41698-022-00313-4 (PMC9547066; doi:10.1038/s41698-022-00313-4)
Supplement: Supplementary file 3 — REPORTING SUMMARY [file 41698_2022_313_MOESM3_ESM.pdf]

nature portfolio

Corresponding author(s): Frank Gonzalez
Thomas Velenosi

Last updated by author(s): Jul 25, 2022

Reporting Summary

Nature Portfolio wishes to improve the reproducibility of the work that we publish. This form provides structure for consistency and transparency in reporting. For further information on Nature Portfolio policies, see our Editorial Policies and the Editorial Policy Checklist.

Statistics

For all statistical analyses, confirm that the following items are present in the figure legend, table legend, main text, or Methods section.

n/a	Confirmed	
☐	☒	The exact sample size (n) for each experimental group/condition, given as a discrete number and unit of measurement
☐	☒	A statement on whether measurements were taken from distinct samples or whether the same sample was measured repeatedly
☐	☒	The statistical test(s) used AND whether they are one- or two-sided<br>*Only common tests should be described solely by name; describe more complex techniques in the Methods section.*
☐	☒	A description of all covariates tested
☐	☒	A description of any assumptions or corrections, such as tests of normality and adjustment for multiple comparisons
☐	☒	A full description of the statistical parameters including central tendency (e.g. means) or other basic estimates (e.g. regression coefficient) AND variation (e.g. standard deviation) or associated estimates of uncertainty (e.g. confidence intervals)
☐	☒	For null hypothesis testing, the test statistic (e.g. F, t, r) with confidence intervals, effect sizes, degrees of freedom and P value noted<br>*Give P values as exact values whenever suitable.*
☒	☐	For Bayesian analysis, information on the choice of priors and Markov chain Monte Carlo settings
☐	☒	For hierarchical and complex designs, identification of the appropriate level for tests and full reporting of outcomes
☒	☐	Estimates of effect sizes (e.g. Cohen's d, Pearson's r), indicating how they were calculated

Our web collection on statistics for biologists contains articles on many of the points above.

Software and code

Policy information about availability of computer code

Data collection	Masslynx 4.2 was used to collect all mass spectrometry data
Data analysis	Data in this study were analyzed using Progenesis QI (Waters), Targetlynx (Waters), R (version 3.6.2), SIMCA 15 (Umetrics), GraphPad Prism versions 8 and 9 (GraphPad Software), MetaboAnalyst 3.0, IsoCorrectoR

For manuscripts utilizing custom algorithms or software that are central to the research but not yet described in published literature, software must be made available to editors and reviewers. We strongly encourage code deposition in a community repository (e.g. GitHub). See the Nature Portfolio guidelines for submitting code & software for further information.

Data

Policy information about availability of data

All manuscripts must include a data availability statement. This statement should provide the following information, where applicable:

- Accession codes, unique identifiers, or web links for publicly available datasets
- A description of any restrictions on data availability
- For clinical datasets or third party data, please ensure that the statement adheres to our policy

The data that support the findings of this study are available from the corresponding authors upon request.

Field-specific reporting

Please select the one below that is the best fit for your research. If you are not sure, read the appropriate sections before making your selection.

☒ Life sciences ☐ Behavioural & social sciences ☐ Ecological, evolutionary & environmental sciences

For a reference copy of the document with all sections, see nature.com/documents/nr-reporting-summary-flat.pdf

Life sciences study design

All studies must disclose on these points even when the disclosure is negative.

Sample size	Sample size for TNBC-PDX in vivo studies was determined using tumor growth information provided by Jackson Labs, the source of all TNBC-PDX models in this study. Biological replicates for ex vivo and in vitro studies were determined experimentally.
Data exclusions	None
Replication	All attempts at replication were successful
Randomization	TNBC-PDX mice were randomized and placed into control or treatment groups for all in vivo studies.
Blinding	The investigator performing analysis of clinical tissue and urine samples was blinded to sample groups. In TNBC-PDX studies, the same investigator maintaining the mice also performed experiments and was therefore, not blinded.

Reporting for specific materials, systems and methods

We require information from authors about some types of materials, experimental systems and methods used in many studies. Here, indicate whether each material, system or method listed is relevant to your study. If you are not sure if a list item applies to your research, read the appropriate section before selecting a response.

Materials & experimental systems

n/a	Involved in the study	
☒	☐	Antibodies
☐	☒	Eukaryotic cell lines
☒	☐	Palaeontology and archaeology
☐	☒	Animals and other organisms
☐	☒	Human research participants
☒	☐	Clinical data
☒	☐	Dual use research of concern

Methods

n/a	Involved in the study	
☒	☐	ChIP-seq
☒	☐	Flow cytometry
☒	☐	MRI-based neuroimaging

Eukaryotic cell lines

Policy information about cell lines

Cell line source(s)	All cell lines were obtained from the NCI Developmental Therapeutics Program (DTP) repository
Authentication	The NCI-DTP authenticates cell lines using the Applied Biosystems AmpFISTR Identifiler testing with PCR amplification.
Mycoplasma contamination	Cells tested negative for mycoplasma contamination.
Commonly misidentified lines (See ICLAC register)	No commonly misidentified cell lines were used in the study

Animals and other organisms

Policy information about studies involving animals; ARRIVE guidelines recommended for reporting animal research

Laboratory animals	Female NSG mice were obtained from NCI Frederick or Jackson Labs at 5 weeks of age and maintained in the NCI animal facility. Mice were housed under 12 hour light cycle conditions and provided water and food ad libitum. TNBC-PDX tumors were subcutaneously implanted when mice were 5-8 weeks old.
Wild animals	None

Field-collected samples	None
Ethics oversight	All animal studies were approved by the NCI Institutional Animal Care and Use Committee (IACUC).

Note that full information on the approval of the study protocol must also be provided in the manuscript.

Human research participants

Policy information about studies involving human research participants

Population characteristics	Stored fresh-frozen tumor samples were obtained from unselected breast cancer patients at all disease stages (PMID:24316975). Stored urine samples were provided from breast cancer patients and/or healthy subjects (PMID:16755295).
Recruitment	Breast cancer patients providing tumor samples were recruited between February 15, 1993, and August 27, 2003, under the NCI resource contract "Collection and Evaluation of Human Tissues and Cells from Donors with an Epidemiology Profile". Dr. Montserrat Garcia-Closas and Dr. Thomas U. Ahearn provided access to data and urine samples from the Polish Breast Cancer Study (NCT00341458). Participants in this study were recruited between 2000 and 2003 and samples were randomly selected.
Ethics oversight	Previous ethics approval described in PMID:24316975 and PMID:16755295 defining usage of stored samples allowed for their use this study.

Note that full information on the approval of the study protocol must also be provided in the manuscript.
